# Prenatal detection of pure proximal 6q14.1 microduplication encompassing *LCA5* gene: A variant of likely benign

**DOI:** 10.1097/MD.0000000000029369

**Published:** 2022-06-17

**Authors:** Fagui Yue, Hongguo Zhang, Lili Luo, Ruizhi Liu, Jili Jing

**Affiliations:** a Center for Reproductive Medicine, Center for Prenatal Diagnosis, First Hospital, Jilin University, Changchun, China; b Jilin Engineering Research Center for Reproductive Medicine and Genetics, Jilin University, Changchun, China.

**Keywords:** 6q14.1 duplication, *LCA5*, prenatal diagnosis, variants of likely benign

## Abstract

Trisomy 6q is a recognizable syndrome which exhibits psychomotor/growth retardation, developmental/intellectual disabilities, feeding difficulties, facial dysmorphism, hearing loss, brain and heart malformations. The purpose of this study was to delineate the prenatal features of proximal 6q14.1 duplication in fetal period, which was rarely reported in clinic. Eight pregnant women who opted for amniocentesis due to the fetal ultrasound abnormalities, maternal serum screening or other indications for prenatal diagnosis between 2019 and 2020. Chromosomal microarray analysis and G-banding analysis were offered after informed consents were obtained. Cytogenetic prenatal investigation showed all fetuses presented normal karyotypes except case 4 exhibiting a balanced chromosomal translocation 46,XX,t (4;8)(p16;q24). The chromosomal microarray analysis detected 0.211–0.242 Mb duplications of 6q14.1 (chr6: 80109532–80351666, hg19) in all 8 cases, encompassing the morbid gene *LCA5* in common. Seven pregnant women (P1-P7) continued their pregnancies and delivered healthy infants at term while the parents of case 8 opted for termination of pregnancy for severe abnormal ultrasound findings. Overall, all neonates were in a good healthy condition with no evident anomalies, ranging from 2 m to 16 m. It is proposed that 6q14.1 duplication involving *LCA5* gene detected in our study might be variants of likely benign. However, further large-scale studies should be gathered to assess its pathogenicity. To our knowledge, our study is the first report focusing on prenatally detected proximal 6q14.1 duplication, accompanied by detailed clinic phenotypes. Diverse ultrasound findings were observed in these cases, ranging from normal to abnormal. More evidence should be gathered to interpret the prenatal genotype-phenotype correlation of 6q14.1 duplication. For these cases with 6q14.1 microduplication, long term follow up should be carried out in case abnormal clinical symptoms or developmental-behavioral disorders emerge.

## 1 Introduction

Trisomy of the long arm of chromosome 6 is a rare chromosomal anomaly, which is regarded as a distinct and recognizable syndrome.^[1]^ This genetic disorder is usually identified at birth or in early childhood.^[2]^ Since the first trisomy 6q case was documented in 1969, more than 40 cases have been described in clinic.^[3,4]^ As a well-defined syndrome, patients of 6q duplication usually presented distinct phenotypes, including mental/growth retardation, low birth weight, short stature, feeding difficulties, microcephaly, prominent forehead, short webbed neck, downslanting palpebral fissures, flat nasal bridge, micrognathia, joint contractures, hypertelorism, carp mouth, heart malformations, brain anomalies, hearing loss, club feet, abnormal genitourinary system and so on.^[5–7]^

Most trisomy 6q cases resulted from the abnormal segregation of a parental balanced translocation, which would usually lead to chromosomal 6q terminal duplication accompanied by another chromosomal deletion. The co-existence of these 2 types of chromosomal anomalies, especially the monosomy of another chromosome, would usually make it complicated to establish a clear interpretation for the genotype-phenotype correlation of pure 6q duplication.^[8–10]^ In addition, pure interstitial and proximal 6q duplications were rarely reported, which also caused difficulty in delineating clinical phenotypes.^[3,5]^ In most trisomy 6q cases, the breakpoints were often located between 6q21 and 6q26, encompassing the 6q27 region.^[8,11]^

The reports on prenatal 6q microduplication are rare, especially lacking of intrauterine phenotypic features. Herein, we delineate prenatal features of proximal 6q14.1 duplication in fetal period and follow up their developmental conditions after birth.

## 2 Materials and methods

### 2.1 Subjects

A total of 8 cases of 6q14.1 duplication diagnosed prenatally between 2019 and 2020 were enrolled in our study. The pregnant women opted for invasive prenatal diagnosis due to abnormal ultrasound findings, maternal serum screening inferring trisomy 21 or others indications. The detailed information of their medical records was listed in Table 1, and the clinical data included the indication for amniocentesis, sex of the fetus, pregnancy history, gestational age, chromosomal microarray analysis results, sonographic findings, the pregnancy outcome and so on. In our study, all parents were healthy, anonymous, and nonconsanguineous without congenital malformations. They denied any exposure to alcohol, teratogenic agents, irradiation, or infectious diseases during their pregnancies. Our study protocol was approved by the Ethics Committee of the First Hospital of Jilin University (No. 2017-397), and the written informed consents were obtained from all couples for publication of this case report and accompanying images.

**Table 1:** Summary of the cytogenetic, chromosomal microarray analysis, and clinical findings of our cases with 6q14.1 duplication.

Case No.	1	2	3	4	5	6	7	8
Sex	M	M	M	F	F	M	F	M
Maternal age	31	25	30	32	24	38	28	35
Pregnancy history	G1P0	G2P1	G2P1	G3P1	G2P1	G2P1	G4P2	G1P0
Gestational age	38w	40w	39w3d	38w3d	40w	38w6d	39w	TOP at 29w
Age at investigation	16m	11m	6.5m	7m	13m	4m	2m	N.A.
Weight(kg)								
birth	N.A.	3.7	3	3.3	3.6	3.5	3.05	N.A.
at evaluation	N.A.	11.5	9	10	10	7.5	6	N.A.
Length(cm)								
birth	N.A.	51	49	50	50	50	50	N.A.
at evaluation	N.A.	80	70	80	75	66	60	N.A.
Karyotypic results	46,XY	46,XY	46,XY	46,XX,t(4;8)(p16;q24)	46,XX	46,XY	46,XX	46,XY
Parental karyotypes	Father: 46,XYMother: 46,XX	Father:46,XYMother: 46,XX	N.A.	N.A.	N.A.	N.A.	N.A.	N.A.
CMA results (hg19)	6q14.1(80121193-803,51666)×3	6q14.1(80121193–803,51666)×3	6q14.1(80109532–803,51666) × 3	6q14.1(80121193–803,51666) × 4Xp22.2(15119891–15524313) × 3	6q14.1(80109532–803,51666) × 37q11.21(64612879–65148399) × 1	6q14.1(80121193–803,32287) × 3	6q14.1(80121193–803,51666) × 3	6q14.1(80121193–803,32287) × 3
Dup/del size(Mb)	0.23Mb	0.23Mb	0.242Mb	0.230Mb0.404Mb	0.242Mb0.536Mb	0.211Mb	0.230Mb	0.211Mb
Inheritance	N.A.	N.A.	pat	N.A.	N.A.	N.A.	N.A.	N.A.
Duplicated gene in 6q14.1	LCA5; SH3BGRL2	LCA5; SH3BGRL2	LCA5; SH3BGRL2	LCA5; SH3BGRL2	LCA5; SH3BGRL2	LCA5	LCA5; SH3BGRL2	LCA5
Indications/reasons for prenatal diagnosis	Fetal nuchal fold (NF) thickness 0.69cm; circulor of umbilical cord	Increased nuchal translucency (NT) thickness	Ultrasounic examination at 30 wks: total situs inversus; mirror-image dextrocardia; transection of inferior vena cava; circulor of umbilical cord	Abnormal childbearing history: heart malformation child; ultrasound findings at 29 wks: nasal bone hypoplasia; circulor of umbilical cord	Abnormal childbearing history: cerebral palsy child; no ultrasound abnormalities	Advanced maternal age; no ultrasound abnormalities	Increased risk for fetal trisomy 21: 1/35; no ultrasound abnormalities	Advanced maternal age; Ultrasounic examination at 23 weeks: ventricular septal defect; persistent right umbilical vein; left ventricular apical thin point
Follow-up outcome	Live birth; no evident anomalies	Live birth; no evident anomalies	Live birth; no evident anomalies	Live birth; no evident anomalies	Live birth; no evident anomalies	Live birth; no evident anomalies	Live birth; no evident anomalies	TOP

CMA = chromosomal microarray analysis, d = days, G = gravida, m = months, N.A. = not available, P = para, pat = paternal inherited, TOP = termination of pregnancy, w = weeks.

### 2.2 Cytogenetic analysis

Cytogenetic analysis was performed using the Giemsa banding technique at a resolution of 300 to 400 bands according to our previous study.^[12]^ The samples were prepared from cultured amniotic fluid cells and peripheral blood cells according to standard protocols. Twenty metaphases were analyzed for all samples. The International System for Human Cytogenetic Nomenclature was used to describe the karyotypes.

### 2.3 Chromosomal microarray analysis

10 mL uncultured amniotic fluid cells was collected through amniocentesis from all pregnant women after following written consent. 5 mL of peripheral blood was collected using a standard vacuum extraction blood-collecting system containing EDTA and heparin for the parents who intended to verify. Then genomic DNA was isolated from using Qiagen micro kit with the manufacturer's protocol. The procedures were conducted through CytoScan 750K array (Affymetrix, Santa Clara, CA, USA), in accordance with manufacturer's protocol and our previous study. ^[13]^ The procedure included genomic DNA extraction, digestion and ligation, PCR amplification, PCR product purification, quantification and fragmentation, labeling, array hybridization, washing and scanning. Thresholds for genome-wide screening were set at ≥200 kb for gains, ≥100 kb for losses. The image data were analyzed using Illumina's Genome Studio software. The final results were interpreted using public databases including database of genomic variants (DGV) (http://www.ncbi.nlm.nih.gov/dbvar/), online mendelian inheritance in man (OMIM) (http://www.ncbi.nlm.nih.gov/omim), DECIPHER (http://decipher.sanger.ac.uk/), ISCA(https://www.iscaconsortium.org/) and PubMed(https://pubmed.ncbi.nlm.nih.gov/). Genomic positions refer to the Human Genome February 2009 assembly (GRCh37/hg19).

## 3 Results

### 3.1 Case 1

A 31-year-old, gravida 1, para 0, pregnant woman underwent amniocentesis for cytogenetic analysis and CMA detection due to increased nuchal fold (NF) thickness and circular of umbilical cord. G-banding analysis showed that the karyotype of the fetus was 46,XY, but CMA revealed a 0.23 Mb duplication in the region of 6q14.1. The parents of the fetus had normal karyotypes. The couple chose to continue the pregnancy according to genetic counseling and delivered a male infant at 38 weeks gestation.

### 3.2 Case 2

A 25-year-old, gravida 2, para 1, pregnant woman underwent amniocentesis for cytogenetic analysis and CMA detection due to increased nuchal translucency (NT) thickness. G-banding analysis showed that the karyotype of the fetus was 46,XY, and CMA revealed a 0.23Mb duplication in the region of 6q14.1. The parents of the fetus presented normal karyotypes. The couple continued the pregnancy according to genetic counseling and delivered a male infant at 40 weeks gestation, whose birth weight was 3700 g and length was 51 cm.

### 3.3 Case 3

A 30-year-old, gravida 2, para 1, pregnant woman underwent ultrasound examination at 30 weeks of gestation, which manifested total situs inversus, mirror-image dextrocardia, transection of inferior vena cava and circular of umbilical cord in the fetus. Afterwards, the woman accepted amniocentesis for cytogenetic analysis and CMA detection. The karyotype of the fetus was identified as 46,XY. However, CMA detected a 0.242 Mb duplication in the region of 6q14.1, which was inherited from the father with normal phenotypes. According to genetic counselling, the couple continued the pregnancy and delivered a male infant at 39w+3d gestation, whose birth weight was 3000 g and length was 49 cm.

### 3.4 Case 4

A 32-year-old, gravida 3, para 1, pregnant woman opted for amniocentesis for cytogenetic analysis and CMA detection due to abnormal childbearing history and abnormal ultrasound findings at 29 weeks. The karyotype of the fetus was 46,XX,t (4;8) (p16;q24). Then the CMA detected a 0.230 Mb duplication in the region of 6q14.1 and a 0.404Mb duplication in the region of Xp22.2. The couple declined CMA verification and continued the pregnancy and delivered a male infant at 38w+3d gestation, whose birth weight was 3300 g and length was 50 cm.

### 3.5 Case 5

A 24-year-old, gravida 2, para 1, pregnant woman opted for amniocentesis for cytogenetic analysis and CMA detection due to childbearing history of cerebral palsy child. The karyotype of the fetus was 46,XX. Then the CMA detected a 0.242 Mb duplication in the region of 6q14.1 and a 0.536 Mb deletion in the region of 7q11.21. The couple chose to continue the pregnancy and delivered a male infant at 40 weeks gestation, whose birth weight was 3600 g and length was 50 cm.

### 3.6 Case 6

A 38-year-old, gravida 2, para 1, pregnant woman accepted amniocentesis for cytogenetic analysis and CMA detection due to advanced maternal age. No ultrasound findings were observed throughout her pregnancy. The karyotype of the fetus was 46,XY. Then the CMA detected a 0.211 Mb duplication in the region of 6q14.1. The couple continued the pregnancy and delivered a male infant at 38w+6d gestation, whose birth weight was 3500 g and length was 50 cm.

### 3.7 Case 7

A 28-year-old, gravida 4, para 2, pregnant woman underwent amniocentesis for cytogenetic analysis and CMA detection due to the high risk of maternal serum screening for Down syndrome. No ultrasound findings were observed throughout her pregnancy. The karyotype of the fetus was 46,XX. Then the CMA detected a 0.230 Mb duplication in the region of 6q14.1. The couple refused the CMA to confirm the chromosomal origin of the fetus and continued the pregnancy. The pregnant woman finally delivered a female infant at 39 weeks gestation, whose birth weight was 3,050 g and length was 50 cm.

### 3.8 Case 8

A 35-year-old, gravida 1, para 0, pregnant woman underwent ultrasound examination at 23 weeks of gestation, which showed ventricular septal defect, persistent right umbilical vein and left ventricular apical thin point. Afterwards she underwent amniocentesis for cytogenetic analysis and CMA detection. G-banding analysis showed that the karyotype of the fetus was 46,XY, and CMA revealed a 0.211 Mb duplication in the region of 6q14.1. Based upon genetic counselling, the couple finally chose to terminate the pregnancy at 29 weeks gestation.

A follow-up on the postnatal health conditions was carried out, mainly including congenital defects, developmental retardation, body stature, craniofacial dysmorphisms and skeletal anomalies. Cases (P1-P7) were in healthy conditions, and no apparent abnormalities were observed till this writing, but long term follow up analysis was still necessary.

## 4 Discussion

We described 8 prenatal cases with pure proximal 6q14.1 duplication at a molecular level for the first time. Their prenatal ultrasound findings varied each other, ranging from normal to abnormal. Compared with terminal duplication, proximal and interstitial duplications in 6q are more uncommon, few of which encompassed the 6q14 region.

Chromosomal 6q duplications are associated with a wide range of clinic manifestations, characterized by psychomotor/growth retardation, developmental/intellectual disabilities, feeding difficulties, facial dysmorphism, hearing loss, brain and heart malformations.^[7,8]^ The duplications involving 6q21-q23, 6q25-6qter and 6q27-qter are recognized as critical regions associated with clinic dysmorphisms.^[14,15]^ Duplications in the regions of q11-q16 and q24-qter are frequently compatible with long-term survival.^[16]^ 6q21q23 duplication is associated with developmental delay, congenital heart defects, depressed nasal bridge, and epicanthal folds.^[9]^ As an imprinted region, 6q24 duplication due to the paternal inheritance is associated with transient neonatal diabetes mellitus.^[17]^ It is inferred that different chromosomal breakpoints, position effects, genetic background, incomplete penetrance and imprinting might be associated with the clinic characteristics of 6q duplication to different degrees.

**Figure 1:**
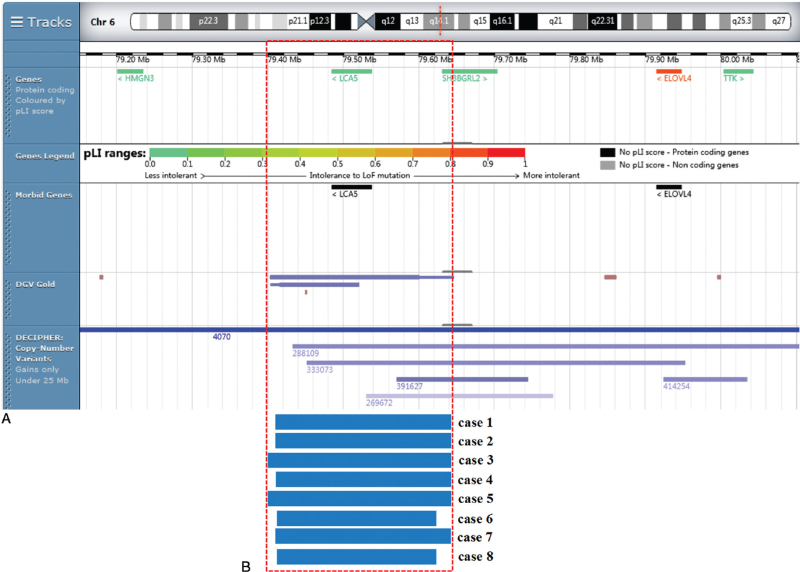
Scale representation of the duplicated region in the long arm of chromosome 6 (https://decipher.sanger.ac.uk/): (A) Location of genes in the region; (B) Duplicated fragments in the present cases encompassing 6q14.1region. The red box indicating the genes located in the 6q14.1 region.

Currently, the genotype-phenotype correlation of proximal 6q duplications remains unclear for lacking of clinic data.^[6]^ To better define the interpretations of 6q14.1 duplication, we summarized duplicated cases involving the 6q14.1 region (Table 2, Fig. 1).^[1,6,7,18–20]^ All duplications involving 6q14.1 varied in size, and were associated with different regions of proximal 6q, locating between 6q11 and 6q21. All cases were postnatally diagnosed and the age ranged from neonate to 14y, presenting different degrees of clinic manifestations. Compared with these cases in the literature, the infants in our study were prenatally diagnosed and at an early age till this writing. Abnormal chromosomal karyotypes were observed in 3 cases (No. 3, 5, and 6), and 2 cases (No. 1 and 2) presented normal karyotypes. As was summarized, 2 cases (No. 1 and 5) had unknown inheritance. The microduplication arose de novo (No. 3) and the remaining 3 cases (No. 2, 4, and 6) carried maternally inherited microduplications. As shown in Table 2, growth/mental retardation and dysmorphic facial features could be observed in 4 cases (No. 3–6). Language retardation (No. 3 and 4) and autism (No. 4 and 5) were also documented. Since these duplicated loci covered not only the 6q14.1 region, it might not be predictive of clinic manifestations for 6q14.1 duplication. Among them, only 2 cases (No. 1 and 2) were involved in pure 6q14.1 duplication. Lu et al^[18]^ described a fetus with 6q14.1 duplication presenting fetal micrognathia while the postnatal examination showed soft cleft palate, with the clinic significance unidentified. Sun et al delineated a novel 622.2 kb 6q14.1 duplication in a female child with congenital solitary kidney, congenital sensorineural hearing loss and cochlear aplasia, encompassing the *MYO6* and *IMPG1* genes.^[7]^ The DGV database included some cases covering this region (2/13759, 0.01%) and no similar duplications were recorded in ClinVar database. Three cases partial overlapping the duplication were recorded in the DECIPHER database: the clinic pathogenicity of 2 cases (DECIPHER 333073 and DECIPHER 269672) was uncertain while the patient (DECIPHER 391627) was likely benign. Based upon the facts above, the 6q14.1 duplication detected in our study might be benign.

**Table 2:** Clinical features of previously published cases spanning6q14.1 duplication.

No.	Sex/age	Duplicated region	Duplicated size	Inheritance	Karyotype	Chromosomal microarray analysis results (hg19)	Clinical manifestations	References
1	n.a./neonate	6q14.1	n.a.	n.a.	46,XN	n.a.	Fetal micrognathia; soft cleft palate; no feeding difficulties	Lu et al ^[18]^
2	F/11 m	6q14.1	622.2 kb	mat	46,XX	6q14.1(76,505,859–77,128,063) × 3	Congenital solitary kidney; congenital sensorineural hearing loss; cochlear aplasia	Sun et al ^[7]^
3	M/14 y	6q11ql5	n.a.	de novo	46, XY, dirdup (6) (q11-q15)	n.a.	Psychomotor/mental retardation; obesity; language retardation; cryptorchidism; hypospadias; small hands with short fingers; flat feet; severe scoliosis; craniofacial dysmorphism: turricephaly with round face, down-slanting eyelids, depressed nasal bridge, short philtrum, macroglossia, narrow high-arched palate and low-set ears	Giardino et al ^[1]^
4	F/8 y	6q13q14.16q14.2q16.1	9.1 Mb13.4 Mb	mat	n.a.	6q13q14.1 (72,415,173–81,550,724) × 36q14.2q16.1(83,904,567–97,318,890) × 3	Mental retardation; development delay; sleep problems; autism; language retardation; sensitivity to noises and light; pes planus of the left foot; muscular hypotonia; syndactyly; dysmorphic facial features: hypertelorism, long broad nasal bridge, small hypoplastic nares, small low set ears, high palate, microcephaly	Wentzel et al ^[20]^ case 1
5	M/8 y	6q14.1q16.1	16.4Mb	n.a.	46,XY, dup (6) (q14.1q16.1)	6q14.1q16.1(78950191–95,395,865) × 3	Developmental delay; intellectual disability; autism spectrum disorder; seizures; hearing loss; immune deficiency; dysmorphic facial features: sparse lateral eyebrows, widely spaced eyes, small ears with overfolded helices, broad nasal bridge, broad and downward pointing nasal tip, elongated columella, short philtrum, thin upper lip	Sanmann et al ^[6]^
6	M/8 y	6q13q21	n.a.	mat	46 XY, −3, +der (3), inv ins (3;6) (p13; q13q21)	n.a.	Growth retardation; mental retardation; joint contractures; bilateral retinal detachment; unusual facies: prominent forehead, flat occiput, prominent pointed chin and multiple unusual hair whorls, downslanting palpebral fissures, hypertelorism, rotated ears, flat nasal bridge, long philtrum, downturned mouth, bifid uvula, cleft palate.	Pierpont et al ^[19]^

F = female, M = male, m = months, n.a. = not available, y = years; genomic coordinates are from the GRCh37/hg19 assembly of the human genome.

Among the detected duplications in our study, 6 cases (cases 1–5, 7) encompassed the *LCA5* and *SH3BGRL2* genes and 2 cases (cases 6 and 8) contained *LCA5* gene only. The *LCA5* gene (OMIM: 611408), containing nine exons, is involved in intraflagellar protein (IFT) transport in photoreceptor cilia. It encodes lebercilin, a ciliary protein which is evolutionary conserved and widely expressed in microtubules, centrosome, and primary cilia. Homozygous mutations of *LCA5* gene are associated with Leber congenital amaurosis, which is prominently an autosomal recessive heterogeneous disorder. As the most severe inherited retinal dystrophies, it is characterized by congenital blindness or severe visual impairment within the first year after birth, nystagmus, sluggish pupillary responses, photophobia, and high hyperopia.^[21,22]^*SH3BGRL2*, known as SH3 domain binding glutamate rich protein like 2, belongs to the SH3BGR family. This gene may be implicated in a wide range of biological functions, such as erythroid differentiation, diabetes, fat intake, nervous system development and intestine formation. In addition, it is also regarded as a tumor suppressor which plays a critical role in clear cell renal cell carcinoma.^[23,24]^ According to the ClinGen database, no available pathogenic evidence for triplosensitivity associated with the 2 genes were recorded. Based upon their functions and implications, it seems that the duplications of *LCA5* and *SH3BGRL2* genes did not have correlation with the prenatal phenotypes of our cases.

In addition, a 0.404 Mb duplication of Xp22.2 for case 4 was detected through CMA. The OMIM genes involved in this region included *ASB9*, *ASB11*, *PIGA*, *PIR*, *FIGF,* and *BMX*. According to DECIPHER database, the mutations of *PIGA* gene may result in paroxysmal nocturnal hemoglobinuria (PNH) and multiple congenital anomalies-hypotonia-seizures syndrome-2(MCAHS2). However, the clinic pathogenicity of the Xp22.2 duplication is still uncertain. A 0.536 Mb deletion of 7q11.21 was detected in case 5, which contained only 1 gene called *ZNF92*. The DGV database included some cases covering this region (119/15799, 0.75%), while no similar clinic data was recorded in the DECIPHER database. It was supposed to be a benign variation. According to the ClinGen database, no available evidence showed that the genes mentioned above were dosage-sensitive.

Currently, the postnatal cases were in healthy states and no other anomalies were observed till this writing. Since they did not exhibit any clinical signs of 6q duplication, it was proposed that the 6q14.1 duplication involving *LCA5* gene detected in our study might be benign. However, long term follow up is still necessary in case abnormal clinical symptoms or developmental-behavioral disorders appear. In addition, 6q14.1 duplication might play a role in the development of children obesity, which also requires attention.^[13]^ For prenatally diagnosed 6q14.1 duplications with various indications, especially advanced maternal age and abnormal serum screening results, the clinicians should take full account of prenatal ultrasound findings, inheritance and incomplete penetrance together to offer genetic counselling for such prenatal cases.

Our study also had some limitations. Not all parents of the fetuses accepted the CMA verification to determine the origins of the 6q14.1 duplications, which might cause some difficulties in genetic counselling. In addition, we only acquired their health conditions in the short term. Whether 6q14.1 duplication would have potential influence on those cases is still unclear, so regular follow-up is necessary.

## 5 Conclusion

As far as we know, this is the first report on pure prenatally detected proximal 6q14.1 duplication, accompanied by detailed clinic phenotypes. Different degrees of ultrasound anomalies were observed in our study while most cases had good postnatal healthy conditions. Owing to scattered distribution of 6q14.1 duplicated loci, the limited research made it challenging to establish a clear genotype-phenotype correlation for 6q14.1 duplication. However, the detected 6q14.1 duplication involving *LCA5* gene in our study might be benign, which would be beneficial for the genetic counselling for such prenatal carriers to some degrees.

## Author contributions

**Conceptualization:** Fagui Yue, Jili Jing

**Data curation:** Hongguo Zhang

**Formal analysis:** Hongguo Zhang

**Investigation:** Lili Luo

**Methodology:** Lili Luo

**Supervision:** Ruizhi Liu

**Validation:** Ruizhi Liu

**Writing – original draft:** Fagui Yue

**Writing – review & editing:** Jili Jing
